# Endothelial damage in septic shock patients as evidenced by circulating syndecan-1, sphingosine-1-phosphate and soluble VE-cadherin: a substudy of ALBIOS

**DOI:** 10.1186/s13054-021-03545-1

**Published:** 2021-03-19

**Authors:** Arianna Piotti, Deborah Novelli, Jennifer Marie Theresia Anna Meessen, Daniela Ferlicca, Sara Coppolecchia, Antonella Marino, Giovanni Salati, Monica Savioli, Giacomo Grasselli, Giacomo Bellani, Antonio Pesenti, Serge Masson, Pietro Caironi, Luciano Gattinoni, Marco Gobbi, Claudia Fracasso, Roberto Latini, the ALBIOS Investigators, Paola Bruzzone, Francesca Pagan, Riccarda Russo, Andrea Confalonieri, Chiara Abbruzzese, Beatrice Vergnano, Stefano Faenza, Antonio Siniscalchi, Elisabetta Pierucci, Andrea Noto, Angelo Pezzi, Paolo Spanu, Vieri Parrini, Roberto Oggioni, Giovanni Stefano Pasetti, Maria Cinzia Casadio, Rosa Buontempo, Sara Carrer, Francesca Piccoli, Tatiana Rizzi, Anselmo Caricato, Monica La Sala, Alessandra Antonaci, Paola Fassini, Silvia Paganini, Virginia Porta, Gabriella Moise, Silvia Marell, Mirella Furia, Maria Cristina Urbano, Roberta Carobbi, Simona Poleni, Hassan Kandil, Andrea Ballotta, Fabrizio Bettini, Manlio Sanseverino, Alessandro Gatta, Francesca Cecchini, Luca Guatteri, Gabriella Ciceri, Ferdinando Raimondi, Roberto Colombo, Sandra Ferraris, Massimo Borelli, Valentina Bellato, Franco Cancellieri, Silvia Senni, Ester Bertocchi, Paola Ferri, Gianpietro Moioli, Andrea Fedele, Alexandra Molin, Giovanni Salati, Pierpaolo Salsi, Emanuela Brunori, Daniele Elisei, Giuseppe Maggio, Federico Guardia Nicola, Marco Cavana, Giacomo Morelli, Arturo Guarino, Michele Isetta, Giorgio Tulli, Valerio Mangani, Nicola Rossi, Marta Ferrari, Francesco Bona, Monica Vay, Teresa Bartoli, Mauro Gallo, Katiuscia Vettoretto, Mauro Della Morte, Enrico Boselli, Daniela Puscio, Monia Bovo, Antonio Galzerano, Manuela Carli, Giovanni Zagara

**Affiliations:** 1 Department of Biochemistry and Molecular Pharmacology, Mario Negri Institute for Pharmacological Research IRCCS, Milan, Italy; 2 Department of Cardiovascular Medicine, Mario Negri Institute for Pharmacological Research IRCCS, Via Mario Negri 2, 20156 Milan, Italy; 3 Emergency Department, Ospedale San Gerardo, Monza, Italy; 4 Anestesia E Rianimazione, ISMETT IRCCS, Palermo, Italy; 5 Anestesia III Terapia Intensiva Adulti, ASST Ospedale Papa Giovanni XXIII, Bergamo, Italy; 6 UOC Anestesia E Rianimazione, IRCCS Arcispedale Santa Maria Nuova, Reggio Emilia, Italy; 7 Dipartimento Di Anestesia, Rianimazione Ed Emergenza Fondazione IRCCS Ca’ Granda Ospedale Maggiore Policlinico, Milan, Italy; 8 Department of Medicine and Surgery, University of Milan-Bicocca, Milan, Italy; 9 Department of Anesthesiology and Critical Care, AOU S. Luigi Gonzaga, Orbassano, Italy; 10 Department of Oncology, Università Degli Studi Di Torino, Turin, Italy; 11 Department of Anesthesiology, Emergency and Intensive Care Medicine, University of Gӧttingen, Gӧttingen, Germany

**Keywords:** Septic shock, Biomarker, Glycocalyx, Syndecan-1, Sphingosine-1-phosphate, VE-cadherin

## Abstract

**Background:**

Septic shock is characterized by breakdown of the endothelial glycocalyx and endothelial damage, contributing to fluid extravasation, organ failure and death. Albumin has shown benefit in septic shock patients. Our aims were: (1) to identify the relations between circulating levels of syndecan-1 (SYN-1), sphingosine-1-phosphate (S1P) (endothelial glycocalyx), and VE-cadherin (endothelial cell junctions), severity of the disease, and survival; (2) to evaluate the effects of albumin supplementation on endothelial dysfunction in patients with septic shock.

**Methods:**

This was a retrospective analysis of a multicenter randomized clinical trial on albumin replacement in severe sepsis or septic shock (the Albumin Italian Outcome Sepsis Trial, ALBIOS). Concentrations of SYN-1, S1P, soluble VE-cadherin and other biomarkers were measured on days 1, 2 and 7 in 375 patients with septic shock surviving up to 7 days after randomization.

**Results:**

Plasma concentrations of SYN-1 and VE-cadherin rose significantly over 7 days. SYN-1 and VE-cadherin were elevated in patients with organ failure, and S1P levels were lower. SYN-1 and VE-cadherin were independently associated with renal replacement therapy requirement during ICU stay, but only SYN-1 predicted its new occurrence. Both SYN-1 and S1P, but not VE-cadherin, predicted incident coagulation failure. Only SYN-1 independently predicted 90-day mortality. Albumin significantly reduced VE-cadherin, by 9.5% (*p* = 0.003) at all three time points.

**Conclusion:**

Circulating components of the endothelial glycocalyx and of the endothelial cell junctions provide insights into severity and progression of septic shock, with special focus on incident coagulation and renal failure. Albumin supplementation lowered circulating VE-cadherin consistently over time.

*Clinical Trial Registration*: ALBIOS ClinicalTrials.gov number NCT00707122.

**Supplementary Information:**

The online version contains supplementary material available at 10.1186/s13054-021-03545-1.

## Background

The glycocalyx is the constituent of the endothelial surface layer which regulates vascular permeability, adhesion of leukocytes and platelets, shear stress and inflammatory processes. The endothelial glycocalyx (eGC) plays an important role in the renal glomerular filtration barrier [1–5].

Many clinical studies support the crucial role of endothelial injury in sepsis-induced organ failure [6–8]. Inflammatory processes such as those occurring in sepsis can severely injure the eGC [9]. Vascular leakage is one of the main clinical problems in sepsis, since it leads to edema, accelerates inflammation, increases platelet aggregation and coagulation. Damage to the eGC is considered an early, sensitive marker of endothelial injury.

Syndecan-1 (SYN-1), a main component of the eGC, is produced by endothelial cells. It is a transmembrane hybrid-type proteoglycan (PG), bearing both heparan sulfate (HS) and chondroitin sulfate (CS) chains, and covers endothelial cells, connecting the inside of the cell with its surroundings. Elevated serum levels of SYN-1, resulting from shedding into the pericellular environment through the action of matrix metalloproteinases (MMPs), may indicate the degradation of eGC [10]. Circulating SYN-1 is therefore considered a biomarker in cardiovascular, inflammatory diseases and tumors [11–16].

Sphingosine 1-Phosphate (S1P) inhibits the degradation of SYN-1 [4]. This circulating sphingolipid has critical roles in the immune and cardiovascular systems [17]. Interacting with the S1P receptors, S1P may inhibit the MMP-dependent shedding of CS, HS, and the SYN-1 ectodomain, thus possibly important in protecting eGC [4]. In addition, S1P can induce synthesis of the glycocalyx’s component, leading to reduced permeability of the endothelium [18]. Thus, both SYN-1 and S1P contribute to maintaining vascular barrier integrity [7, 8, 19], which is severely impaired in sepsis. S1P, which is involved in endothelial cell junction, has been studied in sepsis and negatively correlates with the SOFA score [20]. Several studies in sepsis report that SYN-1 is a biomarker of poor outcomes [11, 21–24].

VE-cadherin is an endothelial transmembrane glycoprotein with a major role in cell–cell adhesion at the adherens junctions. It therefore regulates vascular permeability, which is greatly increased in sepsis [25, 26]. Inflammatory mediators such as TNF-α and cytokines increase in sepsis and promote cleavage of the VE-cadherin extracellular moiety, which then circulates in blood. The concentration of soluble VE-cadherin in plasma may reflect endothelial injury in sepsis [27]. Circulating VE-cadherin has been associated with severe acute kidney injury and organ dysfunction in septic patients [28].

In this study, we aim to assess SYN-1, S1P, and VE-cadherin, their relation with severity of the disease, with other biomarkers, and outcome in septic shock patients.

## Methods

### Study design

ALBIOS was a multicenter, pragmatic, open-label, randomized clinical trial that included 1,818 patients with severe sepsis or septic shock admitted to 100 intensive care units (ICUs). Patients were randomized to receive either albumin and crystalloids or crystalloids alone for the first 28 days after the onset of sepsis or septic shock. Study design, inclusion and exclusion criteria, and the main results have been published elsewhere [29]. The study complied with the 1975 Declaration of Helsinki as revised in 2008 and was approved by the institutional review boards of each center. Written informed consent or deferred consent was obtained from each participant, according to Italian legislation.

### Sample collection

In a subset of 956 patients recruited in 40 centers that participated in a predefined biologic substudy, venous blood samples were serially collected 1, 2, and 7 days after enrolment. Blood samples were collected into ethylenediaminetetraacetic acid (EDTA) tubes, centrifuged, plasma was stored at − 70 °C, and shipped on dry ice to a central repository at − 70 °C until assayed. SYN-1, S1P and VE-cadherin were assayed in all 375 patients with septic shock at study entry had three blood samples available. A cohort of 17 healthy volunteers, blood donors of similar age (between 67 and 77 years) and sex distribution were used as normal reference controls. Expert personnel, blinded to the identity of plasma samples, did all assays.

### Measurements of circulating biomarkers

Total circulating plasma levels of S1P were measured by high-performance liquid chromatography (HPLC) coupled with mass spectrometry (MS). Briefly, human plasma samples (100 µL) to be assayed for S1P were mixed with 5 µL of internal standard (IS, S1P-d7, 5 ng/µL final concentration) and deproteinized by adding 400 µL of methanol. After vortexing, the mixtures were centrifuged at 4 °C for 10 min at 14,000 r.c.f. Supernatants were filtered with 13 mm Acrodisc filters, 0.2 μm PVDF and transferred to an autosampler vial, and 30 μL were injected into the HPLC-MS/MS system (Alliance separation module 2695, Waters, Milford, MA, USA). Chromatographic separation was obtained with an XSelect CSH XP C18 (2.5 μm particle size; 2.1 mm × 50 mm Waters), at a flow rate of 0.3 mL/min. Elution started with 20% of mobile phase A (0.5% formic acid in water) and 80% mobile phase B (0.5% formic acid in methanol) for 1 min, followed by 6 min to 2% in a nonlinear A gradient (curve 4) held for 4 min and a 1-min nonlinear gradient to 20% of A which was maintained for 10 min to equilibrate the column. The total run time was 21 min.

For mass spectrometric analysis we used a Micromass Quattro Micro API triple-quadrupole (Waters, Milford, MA, USA) in positive ion mode and multiple reaction monitoring (MRM) mode, measuring the fragmentation products of the deprotonated pseudo-molecular ions. The choice of fragmentation products for all compounds and the optimization of collision-induced dissociation energies (EC) were selected in continuous-flow mode, using standard solutions at concentrations of 10 ng/µL for all compounds. Data were processed with MassLynx software (Waters, Milford, MA, USA). Plasma concentrations of S1P were quantified by reference to seven-point calibration curves always run in parallel, linear over the concentration ranges (12.5–1000 ng/mL).

Forty-four separate analytical sessions were required for the present study, each with its own calibration curves and quality controls (QC). The 44 calibration curves had a mean R2 of 0.9958 (0.9938–0.9978); the precision of back calculated standards ranged from 1.8 to 14.5% (7.0% for the lower limit of quantitation, LLOQ and 6.3% for the upper limit of quantitation, ULOQ); mean accuracy ranged from 93.1 to 109.2% (99.9% for LLOQ and 104.4% for ULOQ). The quality of the analytical results was checked by assaying quality control samples, which were always within a 20% error.

SYN-1 and VE-cadherin were assayed in plasma by commercial ELISA assay (Synd-1 Human CD138 ELISA Kit, Diaclone, France; Quantikine ELISA Human VE-cadherin, R&D Systems, Minneapolis, USA). Plasma concentrations for SYN-1 were calculated on six-point calibration curves always run in parallel and which were second order polynomial (quadratic) over the used concentration range (8–256 ng/mL). Plasma concentrations of VE-cadherin were calculated on six-point calibration curves always run in parallel, which gave linear regression over the concentration range (1.56–100 ng/mL). The precision of back-calculated standards was 4–15% (15% for the LLOQ and 4% for the ULOQ); mean accuracy was 86–111% (101% for LLOQ and 98% for ULOQ).

Assays of N-terminal pro B-type natriuretic peptide (NT-proBNP), high-sensitive-cardiac troponin T (hs-cTnT) and bio-adrenomedullin (Bio-ADM) are described elsewhere [30, 31].

### Statistical analysis

SYN-1, S1P and VE-cadherin were classified in tertiles, and patients’ characteristics were assessed by using Kruskal–Wallis or Fisher’s exact test, as appropriate. Differences in biomarker concentrations per time point were assessed with the Mann–Whitney test, and the effects of treatment and time by a mixed model including log-transformed values of biomarkers. Kaplan–Meier and Log-Rank analyses were employed to assess differences in survival time between patients categorized according to tertiles of SYN-1, S1P and VE-cadherin. After log-transformation to reach normality, SYN-1, S1P and VE-cadherin were included in Cox proportional hazard models as continuous variables, including adjustment for heart rate, urinary output, SOFA sub-scores (excluding the score included as outcome) and lactate and creatinine at baseline. These survival analyses were done for incident renal replacement therapy (RRT), coagulation failure (SOFA coagulation sub-score 3 or 4) and 90-day mortality as outcome. All analyses were performed with SPSS (SPSS Statistics, IBM version 25).

## Results

Plasma concentrations of SYN-1 and S1P in 17 healthy controls were in accordance with those described in the literature [20, 22]: 73 [50–94] ng/mL and 302 [253.3–404.0], respectively. Plasma concentrations of SYN-1 were more than double in septic shock patients on day 1 (185 [90–381] ng/mL) compared to healthy volunteers (*p* < 0.001), whereas the opposite was true for S1P (*p* < 0.001), with septic shock patients reporting values 3.5 times lower (86.5 [63.7–120.0] ng/mL). Plasma concentrations of VE-cadherin in 17 healthy controls were consistent with those reported by the producer (R&D Systems, Minneapolis USA): 2859 [2394–3750] ng/mL. Concentrations in patients with septic shock were significantly lower (1697 [1313–2199] ng/mL, *p* < 0.001). Baseline characteristics of patients are shown in Additional file 1.

### Syndecan1, S1P, VE-cadherin and clinical characteristics

Table 1 shows the patients’ clinical characteristics by tertiles of SYN-1 on day 1. VE-cadherin increased across tertiles of SYN-1, but S1P did not show any significant trend. SYN-1 was significantly associated with the coagulation (*p* = 0.001), liver (*p* = 0.002) and kidney (*p* = 0.006) SOFA subscales. Heart rate was significantly associated with increased levels of SYN-1 (*p* = 0.001). SYN-1 was significantly different between tertiles of S1P on day 1 (Table 2): levels of SYN-1 in the tertile with the lower S1P levels were 60–90% higher than the higher S1P levels, consistently with an inhibitory effect of S1P on SYN1 shedding. S1P was only associated with the coagulation subscale (*p* = 0.002)*.* For the VE-cadherin tertiles we observed a positive association with SYN-1 at each time point (day 1 *p* < 0.001, day 2 *p* < 0.001, day 7 *p* = 0.04: Table 3). In addition, higher SOFA scores for coagulation (*p* = 0.001) and liver (*p* = 0.011) were associated with higher VE-cadherin. Spearman rank correlations between the biomarkers at each time point are illustrated in Additional file 2: Figure 1.

**Table 1:** Patients’ characteristics by tertiles of syndecan-1 on day 1

SYN-1 (ng/mL)		Lowest tertile range:21–110 (124)	Middle tertile range:111–298 (124)	Highest tertile range:304–2560 (127)	P*
S1P (ng/mL)					
Day 1	Median [IQR]	93 [70–132]	86 [64–119]	82 [62–105]	0.07
Day 2	Median [IQR]	90 [72–131]	90 [64–122]	84 [67–118]	0.48
Day 7	Median [IQR]	97 [70–139]	89 [67–124]	93 [73–130]	0.33
VE-cadherin (ng/mL)					
Day 1	Median [IQR]	1469 [1172–1786]	1729 [1396–2279]	1920 [1549–2489]	1.5 × 10^–8^
Day 2	Median [IQR]	1531 [1247–1969]	1809 [1465–2314]	2072 [1640–2674]	2.1 × 10^–10^
Day 7	Median [IQR]	1708 [1471–2097]	1871 [1523–2315]	2073 [1630–2477]	0.001
Sex					
Female	No. (%)	56 (42.5%)	53 (42.7%)	55 (43.3%)	0.92
Age (year)	Mean ± SD	69.8 ± 13.5	69.0 ± 14.1	67.1 ± 13.9	0.22
BMI (kg/m^2^)	Mean ± SD	26.4 ± 6.2	26.1 ± 5.1	27.5 ± 5.8	0.06
Mean arterial pressure (mmHg)	Mean ± SD	73 ± 12.4	75 ± 13.2	71 ± 13.6	0.13
Heart rate (bpm)	Mean ± SD	101 ± 20.6	104 ± 22.9	111 ± 20.3	0.001
Urine output (mL/h)	Median[IQR]	73 [40–100]	63 [30–100]	50 [20–100]	0.007
Hemoglobin (g/dL)	Mean ± SD	11.1 ± 1.8	10.9 ± 1.7	11.1 ± 1.7	0.69
PaO_2_/FiO_2_	Median[IQR]	214 [143–322]	199 [130–279]	193 [133–266]	0.25
Venous oxygen saturation (%)	Mean ± SD	74.1 ± 8.1	73.1 ± 9.7	73.2 ± 10.3	0.88
Central venous pressure (mmHg)	Mean ± SD	10.4 ± 4.4	11.5 ± 4.9	10.8 ± 4.6	0.16
Ventilatory support needed	No. (%)	108 (87.1%)	109 (87.9%)	115 (90.6%)	0.67
Cardiovascular pathologies	No. (%)	15 (12.1%)	24 (19.4%)	28 (22.0%)	0.11
Hepatic insufficiency	No. (%)	4 (3.2%)	1 (0.8%)	1 (0.8%)	0.21
Immunocompromised	No. (%)	16 (12.9%)	16 (12.9%)	16 (12.6%)	1
Renal insufficiency	No. (%)	0	5 (4.0%)	3 (2.4%)	0.09
COPD	No. (%)	18 (14.5%)	15 (12.1%)	12 (9.4%)	0.47
Baseline SOFA score > 2					
Cardiovascular	No. (%)	124 (100%)	124 (100%)	127 (100%)	-
Coagulation	No. (%)	4 (3.2%)	8 (6.5%)	20 (15.9%)	0.001
Liver	No. (%)	0	2 (1.7%)	9 (7.7%)	0.002
Kidney	No. (%)	19 (15.3%)	25 (20.7%)	40 (32.0%)	0.006
Respiratory	No. (%)	49 (39.5%)	57 (47.5%)	60 (48.0%)	0.32
Dead at 90 days	No. (%)	33 (26.6%)	46 (37.1%)	62 (48.8%)	0.001

SYN-1, syndecan-1; S1P, sphingosine 1-phosphate; IQR, Interquartile range; SD, standard deviation; BMI, body mass index; COPD, chronic obstructive pulmonary disease; SOFA, sequential organ failure assessment

*p value for Kruskal–Wallis or Chi^2^ to compare Syndecan-1 tertiles

**Table 2:** Patients’ characteristics by tertiles of S1P on day 1

S1P (ng/mL)		Lowest tertile range: 15.7–73.2 (124)	Middle tertile range: 73.4–103.3 (124)	Highest tertile range: 103.4–358.0 (127)	p*
SYN-1 (ng/mL)					
Day 1	Median [IQR]	229 [95–401]	222 [95–515]	144 [84–316]	0.02
Day 2	Median [IQR]	547 [287–1157]	427 [241–998]	339 [206–598]	0.002
Day 7	Median [IQR]	650 [346–1086]	511 [254–1027]	337 [242–689]	0.001
VE-cadherin (ng/mL)					
Day 1	Median [IQR]	1674 [1250–2172]	1685 [1338–2239]	1714 [1367–2191]	0.58
Day 2	Median [IQR]	1819 [1489–2330]	1742 [1359–2330]	1706 [1460–2158]	0.79
Day 7	Median [IQR]	1872 [1614–2356]	1860 [1518–2331]	1819 [1506–2319]	0.85
Sex					
Female	No. (%)	60 (48.4%)	56 (45.2%)	48 (37.8%)	0.22
Age (year)	Mean ± SD	67.2 ± 13.9	69.9 ± 14.4	68.9 ± 13.1	0.13
BMI (kg/m^2^)	Mean ± SD	25.8 ± 5.3	27.8 ± 6.6	26.4 ± 5.0	0.04
Mean arterial pressure (mmHg)	Mean ± SD	72.4 ± 14.0	71.7 11.7	74.7 ± 13.4	0.13
Heart rate (bpm)	Mean ± SD	109 ± 22	103 ± 20	103 ± 22	0.03
Urine output (mL/h)	Median[IQR]	60 [30–100]	50 [20–100]	70 [36–120]	0.05
Hemoglobin (g/dL)	Mean ± SD	11.1 ± 1.6	11.0 ± 1.8	11.0 ± 1.8	0.70
PaO_2_/FiO_2_	Median [IQR]	198 [129–281]	201 [130–275]	202 [140–298]	0.41
Venous oxygen saturation (%)	Mean ± SD	73.5 ± 9.5	72.9 ± 10.3	74.1 ± 8.4	0.87
Central venous pressure (mmHg)	Mean ± SD	10.5 ± 7.1	11.1 ± 4.2	11.0 ± 4.9	0.63
Ventilatory support needed	No. (%)	107 (86.3%)	115 (92.7%)	110 (86.6%)	0.20
Cardiovascular pathologies	No. (%)	17 (13.7%)	26 (21.0%)	24 (18.9%)	0.31
Hepatic insufficiency	No. (%)	4 (3.2%)	2 (1.6%)	0	0.13
Immunocompromised	No. (%)	24 (19.4%)	13 (10.5%)	11 (8.7%)	0.03
Renal insufficiency	No. (%)	3 (2.4%)	3 (2.4%)	2 (1.6%)	0.87
COPD	No. (%)	9 (7.3%)	18 (14.5%)	18 (14.2%)	0.14
Baseline SOFA score > 2					
Cardiovascular	No. (%)	124 (100%)	124 (100%)	127 (100%)	-
Coagulation	No. (%)	18 (14.8%)	11 (8.9%)	3 (2.4%)	0.002
Liver	No. (%)	4 (3.5%)	4 (3.3%)	3 (2.5%)	0.88
Kidney	No. (%)	29 (24.2%)	33 (26.6%)	22 (17.5%)	0.20
Respiratory	No. (%)	55 (45.5%)	57 (46.3%)	54 (43.2%)	0.88
Dead at 90 days	No. (%)	45 (36.3%)	50 (40.3%)	46 (36.2%)	0.75

SYN-1, syndecan-1; S1P, sphingosine 1-phosphate; IQR, interquartile range; SD, standard deviation; BMI, body mass index; COPD, chronic obstructive pulmonary disease; SOFA, sequential organ failure assessment

*p value for Kruskal–Wallis or Chi^2^ to compare S1P tertiles

**Table 3:** Patients’ characteristics by tertiles of VE-cadherin on day 1

VE-cadherin (ng/mL)		Lowest tertile range: 528–1475 (125)	Middle tertile range:1483–1985 (123)	Highest tertile range:1988–7487 (127)	P*
SYN-1 (ng/mL)					
Day 1	Median [IQR]	111 [77–262]	191 [87–384]	286 [140–655]	2.5 × 10^–8^
Day 2	Median [IQR]	339 [200–723]	471 [252–820]	550 [287–1470]	4.1 × 10^–5^
Day 7	Median [IQR]	408 [242–763]	459 [283–899]	555 [285–1270]	0.04
S1P (ng/mL)					
Day 1	Median [IQR]	83 [63–116]	87.4 [63.7–119.2]	88.1 [64.0–121.6]	0.72
Day 2	Median [IQR]	82 [62–120]	89.2 [68.0–118.9]	91.7 [74.0–130.5]	0.11
Day 7	Median [IQR]	89 [62–119]	94.2 [72.5–136.2]	92.5 [71.0–139.2]	0.09
Sex					
Female	No. (%)	60 (48.0%)	57 (46.3%)	47 (37.0%)	0.17
Age (year)	Mean ± SD	70.6 ± 12.5	69.1 ± 13.6	66.2 ± 14.9	0.05
BMI (kg/m^2^)	Mean ± SD	26.9 ± 6.5	26.1 ± 4.9	27.0 ± 5.6	0.39
Mean arterial pressure (mmHg)	Mean ± SD	72.9 ± 12.7	74.2 ± 12.6	71.8 ± 13.9	0.20
Heart rate (bpm)	Mean ± SD	104 ± 21	106 ± 25	106 ± 19	0.76
Urine output (mL/h)	Median[IQR]	65 [30–100]	60 [ 30–120]	50 [28–100]	0.55
Hemoglobin (g/dL)	Mean ± SD	11.2 ± 1.8	10.9 ± 1.8	11.0 ± 1.6	0.55
PaO2/FiO2	Median[IQR]	203 [129–267]	200 [132–295]	201 [141–288]	0.71
Venous oxygen saturation (%)	Mean ± SD	72.4 ± 9.7	74.5 ± 8.4	73.6 ± 9.9	0.24
Central venous pressure (mmHg)	Mean ± SD	10.6 ± 4.2	10.8 ± 5.3	11.1 ± 4.5	0.75
Ventilatory support needed	No. (%)	116 (92.8%)	106 (86.2%)	110 (86.6%)	0.19
Cardiovascular pathologies	No. (%)	18 (14.4%)	22 (17.9%)	27 (21.3%)	0.364
Hepatic insufficiency	No. (%)	4 (3.2%)	1 (0.8%)	1 (0.8%)	0.22
Immunocompromised	No. (%)	17 (13.6%)	12 (9.8%)	19 (15.0%)	0.45
Renal Insufficiency	No. (%)	0	2 (1.6%)	6 (4.7%)	0.03
COPD	No. (%)	16 (12.8%)	15 (12.2%)	14 (11.0%)	0.91
Baseline SOFA score > 2					
Cardiovascular	No. (%)	125 (100%)	123 (100%)	127 (100%)	-
Coagulation	No. (%)	3 (2.4%)	9 (7.3%)	20 (15.9%)	0.001
Liver	No. (%)	0	3 (2.6%)	8 (6.7%)	0.01
Kidney	No. (%)	24 (19.4%)	28 (23.1%)	32 (25.6%)	0.50
Respiratory	No. (%)	54 (44.3%)	56 (46.3%)	56 (44.4%)	0.94
Dead at 90 days	No. (%)	40 (32.0%)	43 (35.0%)	58 (45.7%)	0.06

SYN-1, syndecan-1; S1P, sphingosine 1-phosphate; IQR, interquartile range; SD, standard deviation; BMI, body mass index; COPD, chronic obstructive pulmonary disease; SOFA, sequential organ failure assessment

*p value for Kruskal–Wallis or Chi^2^ to compare VE-cadherin tertiles

Lower S1P and higher SYN-1 and VE-cadherin levels were recorded on day 1 in patients with the higher coagulation SOFA-scores at baseline, whereas a better clinical condition (i.e., lower SOFA score) was associated with higher S1P and lower SYN-1 and VE-cadherin levels.

In concordance with this, the proportion of 90-day mortality rose from 26.6% in the lowest tertile of SYN-1 to 48.8% in the highest SYN-1 tertile (*p* = 0.001). S1P levels showed no relation with 90-day mortality. SYN-1 and VE-Cadherin were positively related to NT proBNP and bio-ADM, while S1P negatively, see Additional file 2: Table 1–3.

### Renal replacement therapy (RRT)

**Fig. 1:**
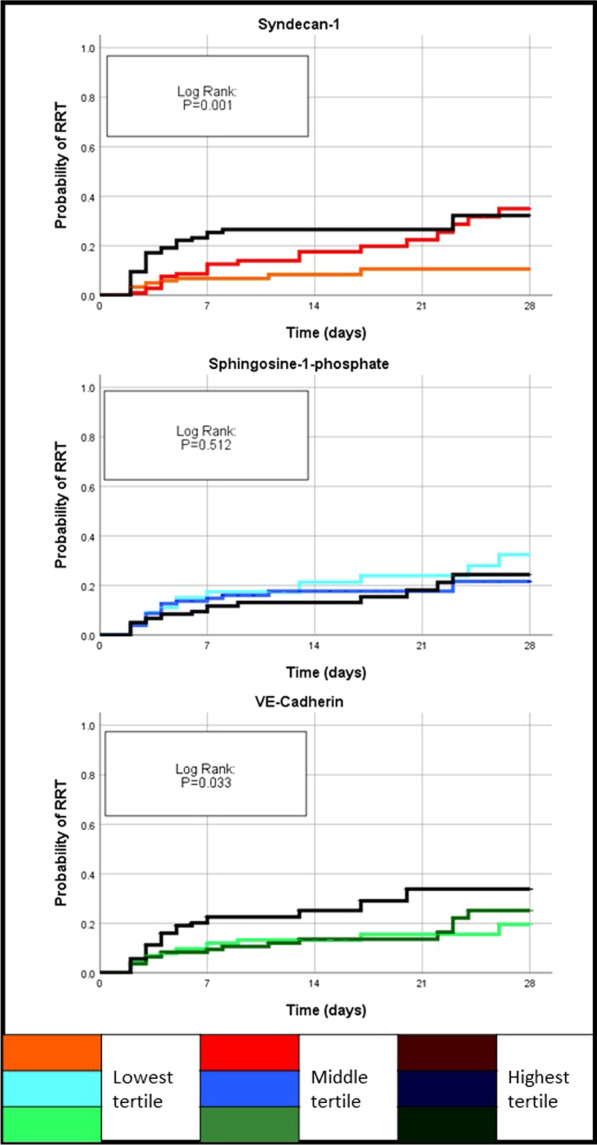
Kaplan–Meier curves for incident renal replacement therapy (RRT). Patients were divided into two groups according to SYN-1, S1P or VE-cadherin concentrations on day 1 by tertiles

The strong association of SYN-1 tertiles with urine output (Table 1) and bio-ADM, a marker of congestion [32] (Additional file 2: Table 1), was also reflected in the significant difference in cumulative RRT incidence (*p* = 0.001) (Fig. 1). With Cox proportional hazard regression analysis relating log-transformed SYN-1 with incident RRT, SYN-1 remained significantly associated after adjusting for study treatment, heart rate, urinary output, lactate, SOFA-sub scores (except kidney) and serum creatinine (HR: 1.98, [95%CI: 1.23–3.18], *p* = 0.005). Tertiles of VE-cadherin were significantly associated with RRT incidence over time (*p* = 0.033, Fig. 1), but not after adjustment. We did not find any association between S1P and RRT.

### SOFA coagulation subscale

**Fig. 2:**
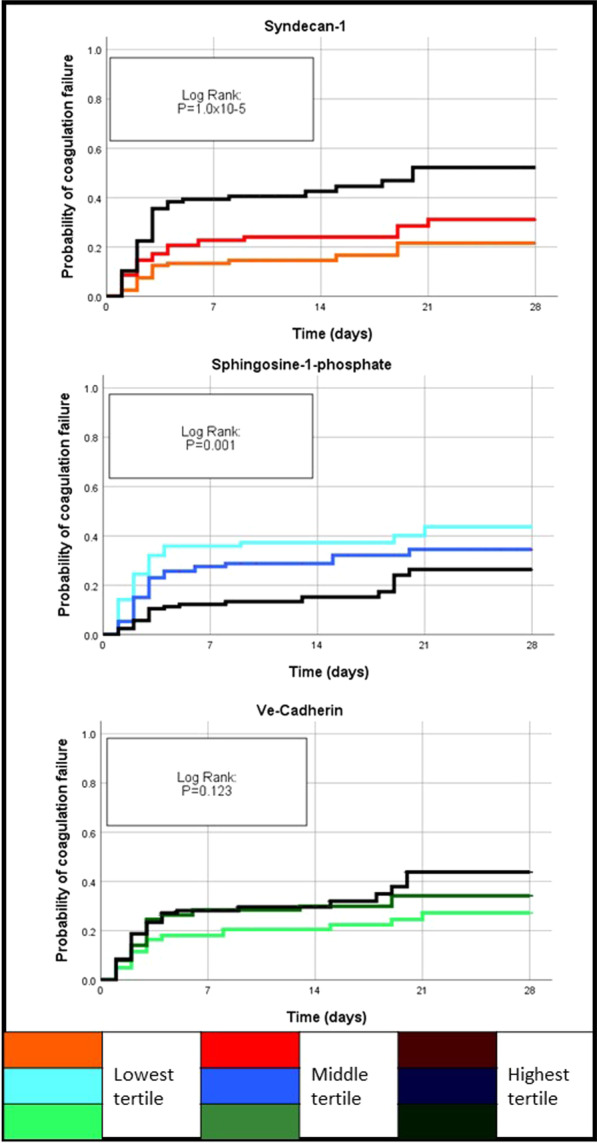
Kaplan–Meier curves for incident coagulation failure (CF). Patients were divided into two groups according to SYN-1, S1P or VE-cadherin concentrations on day 1 by tertile

SOFA Coagulation sub-score was dichotomized into first occurrence of a score of 3 or 4 (“coagulation failure”). Coagulation failure was present in 32 patients at baseline, who were therefore excluded from the analysis. During the first 28 days of follow-up, 98 out of 245 patients experienced coagulation failure. The Kaplan–Meier curves in Fig. 2 show the incidence of coagulation failure in patients with a score of 1 or 2 at baseline, differed across tertiles of SYN-1 and S1P (Log Rank: *p* < 0.001 and *p* = 0.001, respectively). On adjustment [study treatment, heart rate, urinary output, lactate, creatinine, hepatic, respiratory and SOFA sub-scores (except coagulation and kidney)], only SYN-1 and S1P were significantly associated with incident coagulation failure (SYN-1: HR: 1.72, [95%CI: 1.03–2.88], *p* = 0.038; S1P: HR: 0.24, [95%CI: 0.08–0.77], *p* = 0.016).

### Survival

**Fig. 3:**
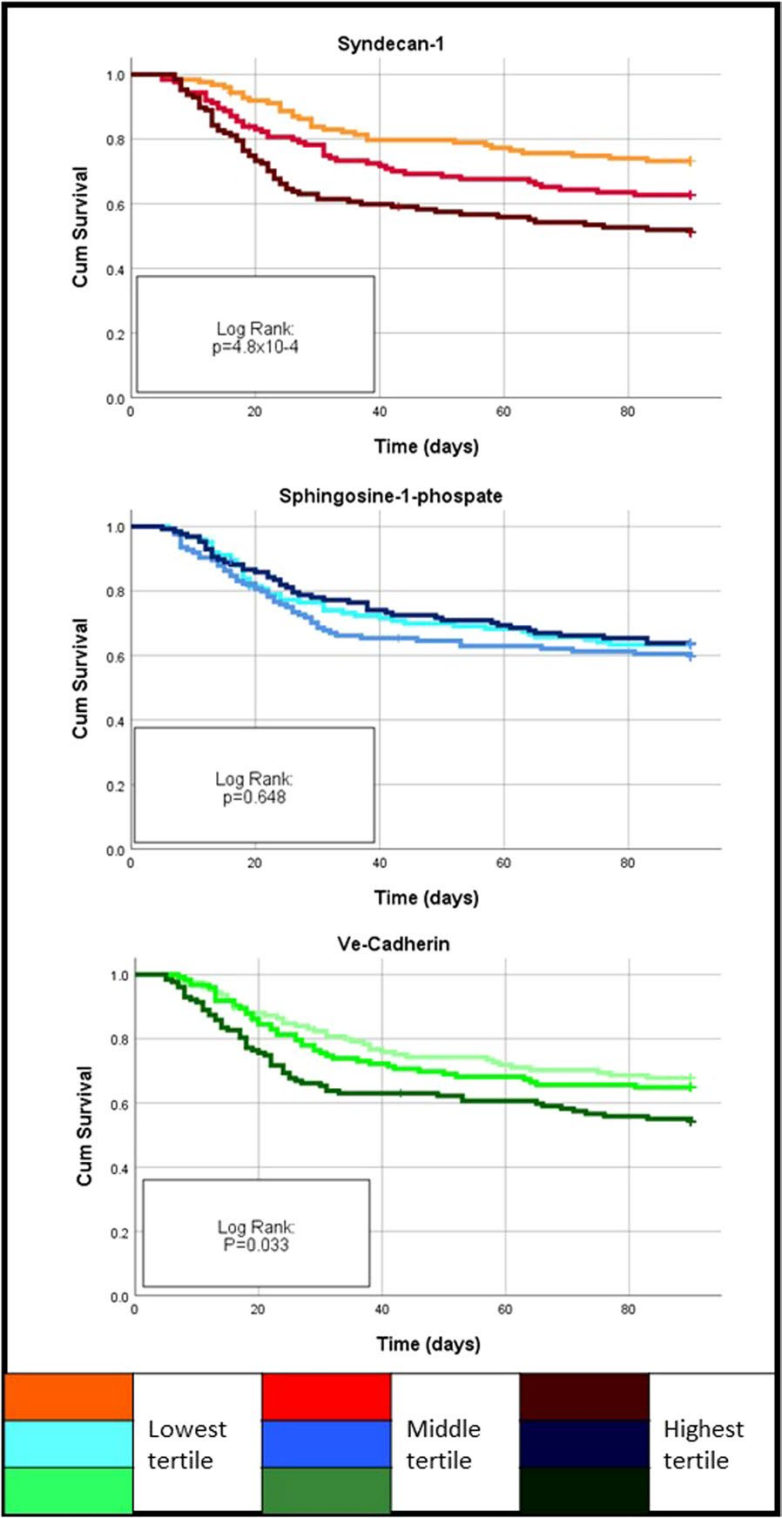
Kaplan–Meier curves for 90-day mortality. Patients were divided into two groups according to SYN-1, S1P or VE-cadherin concentrations on day 1 by tertile

By 90 days of follow-up, 141 (37.6%) of the patients had died, with significant differences in proportions by tertiles of SYN-1 (*p* = 0.001, Table 1). In the Kaplan–Meier curves of Fig. 3, the highest tertiles of SYN-1 and VE-cadherin were significantly associated with lower survival (*p* < 0.001 and *p* = 0.033, respectively). With adjusted Cox proportional hazard survival analysis, SYN-1, but not VE-cadherin and S1P, was associated with 90-day mortality (HR: 1.95 [95%CI: 1.30–2.92].

### Time course and effects of albumin

SYN-1 in septic shock patients rose significantly with time (from 185 [90–381] ng/mL to 481 [264–960] ng/mL, *p* < 0.001) (Table 4). S1P levels showed no trend over time. Concentrations of VE-cadherin rose from day 1 (1697 [1313–2199] ng/mL) to day 7 (1869 [1523–2332] ng/mL, *p* < 0.001) (Table 4).

**Table 4:** SYN-1, S1P and VE-cadherin concentrations over time in 375 patients and by ALBIOS treatment

	ALBIOS patients			*p* value*
	Total (375)	Albumin (188)	Crystalloids (187)	
SYN-1 (ng/mL)				
Day 1	185 [90–381]	176 [86–381]	184 [94–385]	0.41
Day 2	420 [239–962]	400 [228–954]	422 [247–984]	0.29
Day 7	481 [264–960]	469 [255–904]	502 [289–1000]	0.37
Mixed model analysis**		P time		7.6 × 10^–80^
		P treatment		0.253
		P interaction time*treatment		0.883
S1P (ng/mL)				
Day 1	86.5 [63.7–120.0]	87.6 [62.8–123.7]	84.2 [64.0–118.8]	0.91
Day 2	87.2 [67.6–122.1]	90.0 [66.9–128.5]	86.2 [67.7–117.2]	0.35
Day 7	91.6 [69.9–132.9]	93.0 [70.6–138.9]	89.9 [68.0–127.9]	0.29
Mixed model analysis**		P time		0.001
		P treatment		0.147
		P interaction time*treatment		0.717
VE-cadherin (ng/mL)				
Day 1	1697 [1313–2199]	1636 [1270–2068]	1737 [1394–2317]	0.004
Day 2	1751 [1433–2310]	1662 [1381–2144]	1837 [1475–2398]	0.009
Day 7	1869 [1523–2332]	1781 [1494–2251]	1976 [1618–2395]	0.02
Mixed model analysis**		P time		4.5 × 10^–13^
		P treatment		0.003
		P interaction time*treatment		0.193

All biomarker concentrations are reported as median [IQR]

SYN-1, syndecan-1; S1P, sphingosine 1-phosphate; IQR, interquartile range;

*p value for Mann–Whitney to compare ALBIOS-Albumin vs ALBIOS-crystalloids

**p value for mixed model analysis including biomarker value at three time points and treatment. A Greenhouse–Geisser correction was included to correct for sphericity

Fluid replacement with crystalloids supplemented with albumin did not affect SYN-1 and S1P levels, a mixed model analysis showed no significant effects for treatment (Table 4). In contrast, VE-cadherin was significantly affected by treatment, patients with septic shock randomized to albumin had lower VE-cadherin concentrations than the no-albumin group at all three time points (P_treatment_ = 0.003, Table 4). No interaction of time and treatment was found for any of the biomarkers.

## Discussion

In a large representative cohort of patients with septic shock, (1) plasma concentrations of SYN-1 and VE-cadherin were high in those with more severe disease and those of S1P levels were lower; (2) SYN-1, but not S1P and VE-cadherin, predicted the need for RRT; (3) both SYN-1 and S1P, but not VE-cadherin, predicted incident coagulation failure; (4) SYN-1 and, to a lesser extent VE-cadherin, but not S1P, independently predicted 90-day survival; (5) SYN-1 and VE-cadherin rose significantly over the first 7 days; (6) plasma concentrations of soluble VE-cadherin were significantly lower over 7 days in the albumin + crystalloid group than the crystalloid group.

The striking increase of SYN-1 in the first 7 days of follow-up indicates the cumulative insult on the eGC, possibly due to the large fluid load. Capillary leakage is one of the main clinical problems in sepsis, since it leads to edema, accelerates inflammation, increases platelet aggregation and requires large volume of fluids to be infused in order to support organ perfusion and oxygen delivery.

The clinical risk/benefit balance of intravenous fluids in sepsis is still controversial, despite many large clinical trials. On one side, intravenous fluid load can temporarily improve hemodynamics/organ perfusion/O_2_ transport, but on the other, by diffusing into the extravascular space, it may promote ARDS and kidney injury. These hypotheses have been verified in small observational studies [23, 33]. In ALBIOS, we hypothesized that greater endothelial injury would lead to a more positive fluid balance. This is consistent with S1P being inversely related to fluid balance over the first two days after randomization (Additional file 2: Tables 1–3). However, SYN1 levels (unrelated to fluid balance) appear to contradict this. The inverse relationship of S1P with NT-proBNP and bio-ADM, and the direct proportionality of SYN-1 with NT-proBNP and bio-ADM may help understanding the interplay between fluid balance and abnormal vascular permeability. In fact, higher circulating concentrations of bio-ADM were related to more fluid retention (e.g., positive or less negative fluid balance) in septic patients [35].

Circulating soluble VE-cadherin—a major regulator of vascular permeability [24, 25]—was significantly lower in septic patients than in healthy controls. Ostrowski et al*.* measured SYN-1 and VE-cadherin in a small cohort of 20 septic patients, and circulating concentrations of soluble VE-cadherin were also lower in septic patients than in healthy volunteers [34], in agreement with others [26, 38]. In another study, supra-normal concentrations of VE-cadherin have been reported in sepsis [27]. Two tentative explanations can be provided for this controversy: (1) the decrease in circulating VE-cadherin may be the consequence of less VE-cadherin on the cell surface due to internalization under the action of inflammatory cytokines, and/or (2) since VE-cadherin is highly sensitive to lytic enzymes released by activated leukocytes, the 552 amino-acid extracellular moiety which is shed by these enzymes may be further fragmented into smaller molecules which are not recognized by the R&D ELISA polyclonal antibody. Along the same line, a lower expression of VE-cadherin in the lung, compatible with increased permeability, has been reported after LPS injection in mice [36] and in patients with acute respiratory distress syndrome [37].

Higher plasma concentrations of albumin at randomization (i.e., before study treatments were started) suggest a less marked increase in vascular permeability. This hypothesis is supported by the association of higher concentrations of soluble VE-cadherin on day 1 with higher albumin at randomization, an indicator of lower endothelial permeability (Additional file 2: Table 3). The increase in VE-cadherin from day 1 through 7 may indicate a trend toward improvement in vascular permeability in patients surviving; however, the lower concentrations of VE-cadherin in patients receiving crystalloids with albumin do not fit this picture. One possible explanation may be found in the increase in NT-proBNP associated with albumin supplementation, reported in the same patients [30]. Albumin may have contributed to increasing the intravascular volume, due to its oncotic properties compared with crystalloids. The higher retention of fluids in the circulation reflected by higher NT-proBNP, leading to dilution of circulating VE-cadherin, may partially contribute to the effect of albumin.

These results are biologically consistent with the reported anti-inflammatory and antioxidant properties of albumin, possibly leading to endothelial protection [38–40]. In 30 patients with septic shock albumin infusion improved endothelial skin function, possibly through its antioxidant activity [32]. The ALBIOS biological substudy offered an ideal, adequately sized sample of septic patients, for whom serial samples on days 1, 2 and 7 after study inclusion were available. Albumin in plasma acts as a carrier of S1P and this may partially contribute to the protective effect of exogenous albumin in septic shock [41].

SYN-1 and S1P in plasma are inversely related: the higher the S1P, the lower the SYN-1, which is consistent with S1P’s physiological role in stabilizing the endothelial surface layer by inhibiting metalloproteinases responsible for the SYN-1 shedding. Of note is the apparent dissociation between SYN-1 and S1P, where the former is not, but the latter is significantly related to fluid balance, so that higher S1P corresponds to a more favorable fluid balance. Current data do not provide any mechanistic insights into this finding. However, SYN-1, a downhill marker of endothelial integrity consistently seemed a better predictor of outcomes and was more closely related to various markers of septic shock severity. In addition, the strong predictive power of SYN-1 at baseline for RRT confirms its role in protecting the endothelial network, of primary importance in renal glomeruli.

Low concentrations of plasma proteins can injure the endothelial surface layer [42]; thus, supplementation of fluids enriched with albumin may reduce this damage. In ALBIOS, crystalloids supplemented with albumin were not superior to crystalloids alone in fluid replacement in patients with severe sepsis or septic shock. Though a non-prespecified analysis of the main trial showed a significant improvement in survival associated with albumin in patients with septic shock [29]. This suggests that better preservation of the endothelial barrier by albumin supplementation may have contributed to the benefit [40]. Along this same line was the finding that NT-proBNP, a sensitive marker of intravascular volume, was markedly increased by exogenous albumin [30]. However, in the subgroup of 375 patients given albumin mortality was similar to crystalloid group (34.9% vs 37.5%).

The focus on patients with septic shock meant we could not assess the roles played by SYN-1, S1P and VE-cadherin in the progression from severe sepsis to shock. Since we limited our analysis to patients with three blood samples, including day 7, more severely ill patients were lost since they died in the first week. However, we chose to limit our investigation to septic shock since we aimed at assessing whether the beneficial effect of albumin on survival of these patients could be partly explained by circulating SYN-1, S1P or VE-cadherin. The sustained lower concentrations of VE-cadherin, difficult to interpret, together with nonsignificant trends in SYN-1 and S1P and higher levels of NT-proBNP, suggest that albumin may have affected endothelial function.

## Conclusions

In the largest study to date in patients with septic shock, alterations of the eGC and vascular permeability were assessed by assaying in plasma SYN-1, S1P and VE-cadherin, respectively. SYN-1 was directly and S1P inversely related to the severity of the disease. eGC markers of damage predict coagulation failure. Albumin supplementation consistently lowered circulating VE-cadherin over time.

## Supplementary Information

**Additional file 1.** Baseline characteristics of all 375 patients with septic shock included in our analysis.

**Additional file 2:** Syndecan1, Sphingosine-1-phosphate, VE-cadherin and circulating biomarkers. **Table 1.** Circulating biomarkers, albumin and lactate levels by tertiles of Syndecan-1 on day 1; **Table 2.** Circulating biomarkers, albumin and lactate levels by tertiles of S1P on day 1; **Table 3.** Circulating biomarkers, albumin and lactate levels by tertiles of VE-Cadherin on day 1. **Figure 1.** Spearman rank correlation coefficient for SYN-1, S1P and VE-cadherin on days 1, 2 and 7.

## Data Availability

Data supporting this study and the ALBIOS study are not publicly available.

## Abbreviations

ALBIOS: Albumin Italian Outcome Sepsis Trial
AUC: Area under the curve
bio-ADM: Bio-adrenomedullin
eGC: Endothelial glycocalyx
HR: Hazard ratio
NT-proBNP: N-terminal pro B-type natriuretic peptide
RRT: Renal replacement therapy
S1P: Sphingosine-1-phosphate
SOFA: Sequential (sepsis-related) Organ Failure Assessment
SYN-1: Syndecan-1
