# Supplementary material for: Endothelial damage in septic shock patients as evidenced by circulating syndecan-1, sphingosine-1-phosphate and soluble VE-cadherin: a substudy of ALBIOS
Source: Crit Care. 2021 Mar 19;25:113. doi: 10.1186/s13054-021-03545-1 (PMC7980645; doi:10.1186/s13054-021-03545-1)
Supplement: Supplementary file 1 — Additional file 1. Baseline characteristics of all 375 patients with septic shock included in our analysis. [file 13054_2021_3545_MOESM1_ESM.docx]

**Additional file 1**

**Endothelial damage in septic shock patients as evidenced by circulating Syndecan-1, Sphingosine-1-phosphate and soluble VE-cadherin: beneficial effects of albumin supplementation**

Arianna Piotti^1*^, MSc; Deborah Novelli^2*^, PhD; Jennifer Marie Theresia Anna Meessen^2*^, PhD; Daniela Ferlicca^3^, MD; Sara Coppolecchia^4^, MD; Antonella Marino^5^, MD; Giovanni Salati^6^, MD; Monica Savioli^7^, MD; Giacomo Grasselli^7^, MD; Giacomo Bellani^3,8^, MD; Antonio Pesenti^7^, MD; Serge Masson^2^, PhD; Pietro Caironi^9,10^, MD; Luciano Gattinoni^11^, MD; Marco Gobbi^1^, MSc; Claudia Fracasso^1^, MSc; Roberto Latini^2^, MD, on behalf of the ALBIOS Investigators.

* equally contributed

**Affiliations:**
^1^ Department of Biochemistry and Molecular Pharmacology, Mario Negri Institute for Pharmacological Research IRCCS, Milan, Italy;
^2^ Department of Cardiovascular Medicine, Mario Negri Institute for Pharmacological Research IRCCS, Milan, Italy;
^3^ Department of Emergency, Ospedale San Gerardo, Monza, Italy;

^4^ Anestesia e rianimazione, ISMETT IRCCS, Palermo, Italy;

^5^ Anestesia III Terapia Intensiva Adulti, ASST Ospedale Papa Giovanni XXIII, Bergamo, Italy;

^6^ UOC Anestesia e Rianimazione, IRCCS Arcispedale Santa Maria Nuova, Reggio Emilia, Italy;
^7^ Dipartimento di Anestesia, Rianimazione ed Emergenza Fondazione IRCCS Ca' Granda Ospedale Maggiore Policlinico, Milan, Italy;
^8^ Department of Medicine and Surgery, University of Milan-Bicocca, Italy;
^9^ Department of Anesthesiology and Critical Care, AOU S. Luigi Gonzaga, Orbassano, Italy;
^10^ Department of Oncology, Università degli Studi di Torino, Turin, Italy;
^11^ Department of Anaesthesiology, Emergency & Intensive Care Medicine, University of Gӧttingen, Gӧttingen, Germany.

**Corresponding author:**

Roberto Latini
Mario Negri Institute for Pharmacological Research IRCCS

Via Mario Negri 2 20156 Milan, Italy

email: [roberto.latini@marionegri.it](mailto:roberto.latini@marionegri.it)

| **Additional file 1. Baseline characteristics of all 375 patients with septic shock** | | | | |
| --- | --- | --- | --- | --- |
|  | | | | **Total population**  **(375)** |
| SYN-1  (ng/mL) | | Day 1 | Median [IQR] | 185 [90-381] |
|  |  | Day 2 | Median [IQR] | 420 [239-962] |
|  |  | Day 7 | Median [IQR] | 481 [264-960] |
| S1P  (ng/mL) | | Day 1 | Median [IQR] | 86.5 [63.7-120.0] |
|  |  | Day 2 | Median [IQR] | 87.2 [67.6-122.1] |
|  |  | Day 7 | Median [IQR] | 91.6 [69.9-132.9] |
| VE-cadherin (ng/mL) | | Day 1 | Median [IQR] | 1697 [1313-2199] |
|  |  | Day 2 | Median [IQR] | 1751 [1433-2310] |
|  |  | Day 7 | Median [IQR] | 1869 [1523-2332] |
| Sex | | Female | No. (%) | 164 (43.7%) |
| Age | | year | Mean ±SD | 68.6 ± 13.8 |
| BMI | | kg/m2 | Mean ±SD | 26.7 ± 5.7 |
| Mean arterial pressure | | mmHg | Mean ±SD | 73.0 ± 13.1 |
| Heart rate | | bpm | Mean ±SD | 105 ± 22 |
| Urine output | | mL/h | Median[IQR] | 60 [30-100] |
| Hemoglobin | | g/dL | Mean ±SD | 11.0 ± 1.7 |
| PaO_2_/FiO_2_ | | | Median[IQR] | 201 [133-285] |
| Venous oxygen saturation | | % | Mean ±SD | 73.5 ± 9.4 |
| Central venous pressure | | mmHg | Mean ±SD | 10.9 ± 4.7 |
| Ventilatory support needed | | | No. (%) | 332 (88.5%) |
| Cardiovascular pathologies | | | No. (%) | 67 (17.9%) |
| Hepatic insufficiency | | | No. (%) | 6 (1.6%) |
| Immunocompromised | | | No. (%) | 48 (12.8%) |
| Renal insufficiency | | | No. (%) | 8 (2.1%) |
| COPD | | | No. (%) | 45 (12.0%) |
| baseline SOFA score >2 | Cardiovascular | | No. (%) | 375 (100%) |
|  | Coagulation | | No. (%) | 32 (8.5%) |
|  | Liver | | No. (%) | 11 (2.9%) |
|  | Kidney | | No. (%) | 84 (22.4%) |
|  | Respiratory | | No. (%) | 166 (44.3%) |
| Dead at 90 days | | | No. (%) | 141 (37.6%) |
