# Supplementary material for: Endothelial damage in septic shock patients as evidenced by circulating syndecan-1, sphingosine-1-phosphate and soluble VE-cadherin: a substudy of ALBIOS
Source: Crit Care. 2021 Mar 19;25:113. doi: 10.1186/s13054-021-03545-1 (PMC7980645; doi:10.1186/s13054-021-03545-1)
Supplement: Supplementary file 2 — Additional file 2: Syndecan1, Sphingosine-1-phosphate, VE-cadherin and circulating biomarkers. Table 1. Circulating biomarkers, albumin and lactate levels by tertiles of Syndecan-1 on day 1; Table 2. Circulating biomarkers, albumin and lactate levels by tertiles of S1P on day 1; Table 3. Circulating biomarkers, albumin and lactate levels by tertiles of VE-Cadherin on day 1. Figure 1. Spearman rank correlation coefficient for SYN-1, S1P and VE-cadherin on days 1, 2 and 7. [file 13054_2021_3545_MOESM2_ESM.docx]

**Additional file 2**

**Endothelial damage in septic shock patients as evidenced by circulating Syndecan-1, Sphingosine-1-phosphate and soluble VE-cadherin: beneficial effects of albumin supplementation**

Arianna Piotti^1*^, MSc; Deborah Novelli^2*^, PhD; Jennifer Marie Theresia Anna Meessen^2*^, PhD; Daniela Ferlicca^3^, MD; Sara Coppolecchia^4^, MD; Antonella Marino^5^, MD; Giovanni Salati^6^, MD; Monica Savioli^7^, MD; Giacomo Grasselli^7^, MD; Giacomo Bellani^3,8^, MD; Antonio Pesenti^7^, MD; Serge Masson^2^, PhD; Pietro Caironi^9,10^, MD; Luciano Gattinoni^11^, MD; Marco Gobbi^1^, MSc; Claudia Fracasso^1^, MSc; Roberto Latini^2^, MD, on behalf of the ALBIOS Investigators.

* equally contributed

**Affiliations:**
^1^ Department of Biochemistry and Molecular Pharmacology, Mario Negri Institute for Pharmacological Research IRCCS, Milan, Italy;
^2^ Department of Cardiovascular Medicine, Mario Negri Institute for Pharmacological Research IRCCS, Milan, Italy;
^3^ Department of Emergency, Ospedale San Gerardo, Monza, Italy;

^4^ Anestesia e rianimazione, ISMETT IRCCS, Palermo, Italy;

^5^ Anestesia III Terapia Intensiva Adulti, ASST Ospedale Papa Giovanni XXIII, Bergamo, Italy;

^6^ UOC Anestesia e Rianimazione, IRCCS Arcispedale Santa Maria Nuova, Reggio Emilia, Italy;
^7^ Dipartimento di Anestesia, Rianimazione ed Emergenza Fondazione IRCCS Ca' Granda Ospedale Maggiore Policlinico, Milan, Italy;
^8^ Department of Medicine and Surgery, University of Milan-Bicocca, Italy;
^9^ Department of Anesthesiology and Critical Care, AOU S. Luigi Gonzaga, Orbassano, Italy;
^10^ Department of Oncology, Università degli Studi di Torino, Turin, Italy;
^11^ Department of Anaesthesiology, Emergency & Intensive Care Medicine, University of Gӧttingen, Gӧttingen, Germany.

**Corresponding author:**

Roberto Latini
Mario Negri Institute for Pharmacological Research IRCCS

Via Mario Negri 2 20156 Milan, Italy

email: [roberto.latini@marionegri.it](mailto:roberto.latini@marionegri.it)

**Syndecan1, Sphingosine-1-phosphate, VE-cadherin and circulating biomarkers**

In Supplementary Tables 1-3**,** the concentrations of selected cardiovascular biomarkers by tertile of SYN-1, S1P and VE-cadherin are shown. Patients in the highest tertile of SYN-1 and VE-cadherin on day 1 had significantly higher levels of NT-proBNP and troponin T as compared to those in the lowest tertile. NT-proBNP, but not troponin T, was inversely associated with S1P.

SYN-1 and S1P were in opposite direction associated with lactate and bioADM, with a more pronounced difference in SYN-1 as compared to S1P, however we did not see significant differences for VE-cadherin. SYN-1 showed strong, significant association with PTX3 at all three time points, with higher SYN-1 tertiles associated with higher levels of PTX3. Fluid balance was significantly more favorable (i.e. less positive) at higher concentrations of S1P on day 1 and 2 (p=0.043 and p=0.033, respectively). Tertiles of VE-cadherin at day 1 were significantly associated to albumin levels at baseline (i.e. before randomization).

In agreement with the relation with coagulation failure within SOFA, platelet counts were inversely proportional to SYN-1 and directly with S1P at baseline and over the first 7 days (Supplementary Tables 1 and 2). The same trend was evident also for VE-cadherin, though not always statistically significant (Supplementary Table 3).

| Supplementary Table 1. Circulating biomarkers, albumin and lactate levels by tertiles of Syndecan-1 on day 1 | | | | | |
| --- | --- | --- | --- | --- | --- |
| Syndecan-1 (ng/mL)  Median [IQR] | | Lowest tertile range:  21.0-110.1  N=124 | Middle tertile  range:  110.6-298.3  N=124 | Highest tertile  range:  303.6-2560.0  N=127 | P* |
| Lactate (mmol/L) | baseline | 2.35 [1.30-4.00] | 2.37 [1.59-4.37] | 3.65 [2.24-5.80] | 4.0x10^-6^ |
|  | day1 | 1.60 [1.16-2.40] | 1.65 [1.30-2.54] | 2.52 [1.68-4.13] | 1.4x10^-8^ |
|  | day2 | 1.30 [1.00-1.80] | 1.52 [1.20-2.06] | 1.97 [1.49-2.80] | 7.6x10^-10^ |
|  | day7 | 1.20 [1.0-1.50] | 1.50 [1.10-1.90] | 1.40 [1.00-2.00] | 0.008 |
| Albumin  (g/L) | baseline | 23.0 [19.0-28.5] | 24.3 [19.5-29.0] | 24.0 [20.0-29.0] | 0.630 |
|  | day1 | 27.0 [22.0-32.0] | 27.0 [24.1-31.0] | 26.0 [21.0-31.0] | 0.618 |
|  | day2 | 28.0 [22.0-31.0] | 27.0 [24.0-30.0] | 27.0 [23.0-30.8] | 0.836 |
|  | day7 | 28.5 [25.0-31.0] | 27.0 [23.8-29.4] | 27.0 [23.0-30.0] | 0.106 |
| NT-proBNP (ng/L) | day1 | 3420 [1220-7003] | 6137 [2469-20246] | 11319 [4342-28214] | 4.8x10^-10^ |
|  | day2 | 2396 [1012-6229] | 4660 [1759-15950] | 8761 [2917-20469] | 5.9x10^-8^ |
|  | day7 | 1245 [507-2656] | 2242 [776-5831] | 2514 [937-10727] | 4.0x10^-6^ |
| hs cTnT  (ng/L) | day1 | 40.4 [22.6-82.8] | 56.2 [32.7-164.4] | 82.3 [39.4-233.4] | 1.6x10^-5^ |
|  | day2 | 33.9 [21.4-74.3] | 48.8 [26.8-183.9] | 77.3 [33.6-167.8] | 1.1x10^-4^ |
|  | day7 | 28.9 [16.4-58.0] | 43.9 [25.7-110.0] | 55.3[24.8-123.0] | 1.1x10^-4^ |
| Bio ADM (pg/mL) | day1 | 99.4 [57.9-146.1] | 111.6 [72.7-198.3] | 188.8 [104.1-306.4] | 1.3x10^-7^ |
|  | day2 | 72.5 [42.4-136.1] | 91.0 [61.1-171.1] | 142.0 [82.5-242.8] | 1.8x10^-8^ |
|  | day7 | 40.2 [25.1-77.5] | 62.0 [36.2-94.2] | 68.0 [38.7-136.3] | 4.2x10^-5^ |
| PTX3  (ng/mL) | day1 | 54.5 [28.4-155.6] | 79.3 [39.7-183.4] | 136.1 [54.1-384.7] | 8.0x10^-6^ |
|  | day2 | 36.2 [17.5-88.9] | 46.5 [25.5-117.9] | 51.9 [31.2-176.1] | 0.001 |
|  | day7 | 14.8 [8.6-29.3] | 19.0 [9.9-35.5] | 25.1 [14.2-42.8] | 3.0x10^-4^ |
| Fluid administration (mL) | day1 | 4297 [3200-6046] | 4077 [3163-4700] | 4400 [3000-5741] | 0.852 |
|  | day2 | 4095 [3278-5068] | 3700 [3021-4573] | 4050 [3324-5410] | 0.027 |
|  | day7 | 3001 [1649-3899] | 2882 [213-3765] | 3000 [2421-3800] | 0.315 |
| Fluid balance (mL/day) | day1 | 1370 [273-2747] | 1350 [105-2438] | 1340 [122-2850] | 0.610 |
|  | day2 | 650 [-524 – 1582] | 337 [-542 – 1298] | 430 [-795 – 2191] | 0.478 |
|  | day7 | -408 [-1483 – 0] | 0 [-974 – 144] | -630 [-1752 – 0] | 0.018 |
| Platelets  (x10^3^/mm^3^) | baseline | 181 [124-241] | 155 [104-209] | 135 [78-211] | 0.001 |
|  | day 1 | 158 [103-207] | 135 [77-188] | 108 [57-170] | 3.7x10^-4^ |
|  | day 2 | 142 [92-197] | 114 [65-183] | 84 [40-144] | 2.1x10^-7^ |
|  | day 7 | 198 [127-281] | 153 [48-238] | 113 [69-198] | 1.0x10^-6^ |
| * P value for Kruskal-Wallis test. NT-proBNP – N-Terminal B-type natriuretic peptide, hs cTnT – high sensitive cardiac troponin T, bioADM – biologically active adrenomedullin, PTX3 – pentraxin-3. | | | | | |

| Supplementary Table 2. Circulating biomarkers, albumin and lactate levels by tertiles of S1P on day 1 | | | | | |
| --- | --- | --- | --- | --- | --- |
| S1P (ng/mL)  Median [IQR] | | Lowest tertile range:  15.7-73.2  N=124 | Middle tertile  range:  73.4-103.3  N=124 | Highest tertile  range:  103.4-358.0  N=127 | P* |
| Lactate  (mmol/L) | baseline | 2.96[1.90-5.18 ] | 2.80 [1.68-4.50] | 2.38 [1.70-4.00] | 0.107 |
|  | day1 | 2.00 [1.40-3.35] | 2.00 [1.40-3.28] | 1.65 [1.20-2.50] | 0.024 |
|  | day2 | 1.70 [1.20-2.40] | 1.80 [1.27-2.50] | 1.40 [1.00-1.80] | 0.001 |
|  | day7 | 1.37 [1.00-1.90] | 1.40 [1.10-1.80] | 1.30 [1.00-1.60] | 0.109 |
| Albumin  (g/L) | baseline | 25.0 [20.5-29.0] | 23.0 [19.0-28.0] | 23.0 [19.0-29.0] | 0.159 |
|  | day1 | 28.0 [23.0-31.8] | 26.0 [22.0-30.0] | 27.0 [22.0-31.0] | 0.277 |
|  | day2 | 27.0 [23.0-31.0] | 27.0 [24.0-30.0] | 27.0 [22.0-30.0] | 0.756 |
|  | day7 | 28.0 [25.0-30.0] | 27.0 [23.0-30.0] | 28.0 [23.0-31.0] | 0.457 |
| NT-proBNP (ng/L) | day1 | 8356 [2823-25269] | 6049 [2374-16776] | 4358 [1363-11193] | 0.001 |
|  | day2 | 5865 [2238-18826] | 3934 [1487-11085] | 3300 [1143-10794] | 0.006 |
|  | day7 | 2416 [1114-6475] | 1488 [668-3655] | 1785 [595-4421] | 0.028 |
| hs cTnT  (ng/L) | day1 | 58.1 [34.5-149.8] | 57.5 [29.7-153.2] | 51.8 [25.3-145.2] | 0.721 |
|  | day2 | 54.9 [29.8-128.2] | 49.6 [25.5-118.9] | 41.3 [21.1-110.3] | 0.253 |
|  | day7 | 43.6 [24.2-101.7] | 43.4 [22.8-108.9] | 32.6 [16.3-80.2] | 0.190 |
| Bio ADM  (pg/mL) | day1 | 129.0 [85.3-232.0] | 140.7 [84.3-271.9] | 102.3 [59.5-165.7] | 1.6x10^-4^ |
|  | day2 | 109.2 [63.8-209.4] | 112.6 [61.9-204.0] | 77.1 [44.4-145.4] | 4.8x10^-4^ |
|  | day7 | 72.8 [42.4-112.4] | 66.0 [33.5-129.9] | 40.0 [26.4-70.5] | 3.0x10^-6^ |
| PTX3  (ng/mL) | day1 | 118.7 [44.2-225.5] | 79.1 [31.8-200.3] | 88.5 [39.5-210.2] | 0.116 |
|  | day2 | 52.6 [29.4-149.7] | 44.9 [25.0-87.7] | 43.2 [21.7-103.5] | 0.040 |
|  | day7 | 18.9 [11.2-37.1] | 20.1 [10.0-39.1] | 18.1 [8.7-36.0] | 0.321 |
| Fluid administration (mL) | day1 | 440 [3300-6043] | 4317 [3000-5700] | 4100 [3078-5850] | 0.653 |
|  | day2 | 3806 [3144-4685] | 4003 [3300-5268] | 4000 [3200-5000] | 0.344 |
|  | day7 | 2905 [1920-3782] | 3080 [224-3751] | 2970 [765-3957] | 0.883 |
| Fluid balance (mL/day) | day1 | 1733 [536-3145] | 1314 [-89 – 2580] | 1060 [190-2310] | 0.043 |
|  | day2 | 723 [-485 – 1875] | 469 [-761 – 1725] | 290 [-930 – 1215] | 0.033 |
|  | day7 | -200 [-1291 – 66] | -792 [-1695 – 0] | -225 [-1484 – 0] | 0.235 |
| Platelets  (x10^3^/mm^3^) | baseline | 135 [74-207] | 152 [107-223] | 166 [134-253] | 3.3x10^-4^ |
|  | day 1 | 97 [51-170] | 144 [76-179] | 161 [108-242] | 5.3x10^-7^ |
|  | day 2 | 82 [40-155] | 133 [61-169] | 146 [93-208] | 2.0x10^-8^ |
|  | day 7 | 106 [63-192] | 145 [92-239] | 201 [120-291] | 4.5x10^-7^ |
| * P value for Kruskal-Wallis test. S1P – Sphingosine 1-phosphate; NT-proBNP – N-Terminal B-type natriuretic peptide, hs cTnT – high sensitive cardiac troponin T, bioADM – biologically active adrenomedullin, PTX3 – pentraxin-3. | | | | | |

| Supplementary Table 3. Circulating biomarkers, albumin and lactate levels by tertiles of VE-Cadherin on day 1 | | | | | |
| --- | --- | --- | --- | --- | --- |
| VE-cadherin (ng/mL)  Median [IQR] | | Lowest tertile range:  528-1475  N=125 | Middle tertile  Range:  1483-1985  N=123 | Highest tertile  Range:  1988-7487  N=127 | P* |
| Lactate  (mmol/L) | baseline | 2.60 [1.58-4.50] | 2.70 [1.90-4.22] | 2.92 [1.67-4.98] | 0.616 |
|  | day1 | 1.70 [1.20-2.54] | 1.80 [1.30-3.40] | 2.00 [1.40-3.30] | 0.078 |
|  | day2 | 1.50 [1.10-2.10] | 1.60 [1.20-2.20] | 1.70 [1.10-2.50] | 0.264 |
|  | day7 | 1.33 [1.00-1.90] | 1.40 [1.00-1.70] | 1.30 [1.08-1.80] | 0.959 |
| Albumin  (g/L) | baseline | 21.0 [18.0-26.0] | 24.0 [20.0-29.0] | 25.5 [21.0-30.0] | 3.8x10^-4^ |
|  | day1 | 25.0 [22.0-30.0] | 27.0 [22.0-31.0] | 28.0 [24.9-31.6] | 0.033 |
|  | day2 | 27.0 [23.0-30.0] | 27.0 [23.0-29.8] | 28.0 [24.0-31.0] | 0.165 |
|  | day7 | 28.0 [23.0-31.0] | 28.0 [24.0-30.0] | 27.0 [25.0-31.0] | 0.762 |
| NT-proBNP (ng/L) | day1 | 4423 [1220-9607] | 5931 [2359-15764] | 8603 [2806-28778] | 3.5x10^-4^ |
|  | day2 | 3392 [1195-8263] | 4130 [1796-11737] | 7442 [1692-20619] | 0.002 |
|  | day7 | 1488 [610-3428] | 2042 [694-3778] | 2532 [953-10229] | 0.003 |
| hs cTnT  (ng/L) | day1 | 48.6 [30.4-121.0] | 51.1 [25.3-107.1] | 70.6 [40.1-249.5] | 0.002 |
|  | day2 | 41.0 [24.7-106.4] | 38.7 [21.4-90.8] | 69.8 [33.2-167.8] | 0.008 |
|  | day7 | 36.3 [19.6-83.9] | 35.9 [18.9-70.9] | 54.9 [25.7-117.3] | 0.036 |
| Bio ADM  (pg/mL) | day1 | 114.6 [72.8-203.8] | 124.7 [83.0-222.7] | 129.1 [66.1-236.9] | 0.750 |
|  | day2 | 96.2 [58.1-179.0] | 93.2 [52.4-181.4] | 100.5 [52.6-206.8] | 0.915 |
|  | day7 | 62.7 [35.8-98.6] | 55.5 [28.2-100.6] | 56.4 [32.9-107.0] | 0.470 |
| PTX3  (ng/mL) | day1 | 88.8 [37.7-193.1] | 81.2 [35.0-192.1] | 102.8 [31.8-235.7] | 0.790 |
|  | day2 | 47.0 [26.6-115.9] | 42.9 [17.8-117.7] | 41.9 [23.1-104.7] | 0.729 |
|  | day7 | 17.9 [9.1-35.9] | 19.4 [10.0-37.9] | 18.1 [10.0-36.0] | 0.683 |
| Fluid administration (mL) | day1 | 4500 [3000-6140] | 4000 [3128-5700] | 4300 [3200-5775] | 0.627 |
|  | day2 | 3950 [3380-5045] | 4050 [3220-5052] | 3800 [3010-4800] | 0.324 |
|  | day7 | 3186 [2668-3900] | 3345 [2758-4350] | 3152 [2682-4100] | 0.356 |
| Fluid balance (mL/day) | day1 | 1340 [293-2945] | 1350 [-70 – 2590] | 1397 [250-2680] | 0.572 |
|  | day2 | 640 [-523 – 1618] | 290 [-616 – 1500] | 368 [-780 – 1594] | 0.775 |
|  | day7 | -923 [-1826 - -83] | -611 [-1719 – 303] | -740 [-1541 – 297] | 0.379 |
| Platelets  (x10^3^/mm^3^) | baseline | 167 [126-239] | 153 [108-225] | 135 [75-204] | 0.004 |
|  | day 1 | 146 [97-190] | 129 [77-196] | 111 [57-185] | 0.058 |
|  | day 2 | 116 [82-174] | 105 [63-185] | 103 [40-185] | 0.140 |
|  | day 7 | 165 [112-250] | 151 [95-246] | 131 [70-217] | 0.030 |
| * P value for Kruskal-Wallis test. NT-proBNP – N-Terminal B-type natriuretic peptide, hs cTnT – high sensitive cardiac troponin T, bioADM – biologically active adrenomedullin, PTX3 – pentraxin-3. | | | | | |

**Supplementary Figure 1** – Spearman rank correlation coefficient for SYN-1, S1P and VE-cadherin at day 1, day 2 and day 7.
